# Dignity at stake in educational relations - The significance of confirmation

**DOI:** 10.1177/09697330221089083

**Published:** 2022-06-20

**Authors:** Tone Stikholmen, Dagfinn Nåden, Herdis Alvsvåg

**Affiliations:** Centre of Diaconia and Professional Practice, 155312 VID Specialized University, Bergen, Norway; Faculty of Health Sciences, Department of Nursing and Health Promotion, 60499 OsloMet, Oslo, Norway; Faculty of Health Studies, 155312 VID Specialized University, Bergen, Norway

**Keywords:** Dignity, nursing education, nursing students’ experiences, student–supervisor relationship, hermeneutics

## Abstract

**Introduction:**

It is a goal in nursing education to promote students’ dignity and facilitate this core value. Students’ experience of dignity is shaped by the student–supervisor relationship. Literature shows limited knowledge about how nursing students experience their own dignity during education.

**Research aim:**

The aim of the study is to develop an understanding of how nursing students experience their own dignity in relation to supervisors, and what significance these experiences have in education.

**Research design:**

Gadamer’s philosophical hermeneutics was chosen as the approach, and narratives and qualitative interviews were conducted. The interpretation process was inspired by Fleming, Gaidys and Robbs.

**Participants and research context:**

Nineteen nursing students in the final year of their education were included in the study. They represented six different campuses at three different educational institutions. The qualitative interviews took place at the educational institutions.

**Ethical considerations:**

The research recommendations of the Declaration of Helsinki were followed. Access to the students was given by the educational institutions. All interested students signed a continuous informed consent.

**Findings:**

Students’ dignity was at stake in encounters with supervisors during education. Decisive for experience of dignity was the supervisor’s ability to confirm the student through acknowledgment, reassurance and seeing them as individuals. Experienced dignity had a crucial impact on students’ life courage and their ability to be present.

**Discussion:**

The discussion emphasizes the vulnerable dignity of students, the importance of confirmation and the significance perceived dignity has.

**Conclusion:**

Students’ experiences tilted between perceived dignity and offense, and placed students’ dignity in a vulnerable position. Crucial for perceived dignity was the confirmation the students received from their supervisors. Perceived dignity gave the students courage and increased their ability to be present, which provided better opportunities for learning and development.

## Introduction

Dignity is an essential value in nursing.^1,2^ Research on dignity in nursing focuses mainly on patients' dignity and less on nurses' and nursing students' experience of dignity.^3,4^ This article deals with how nursing students experience their own dignity during education, and what significance the experience of dignity has.

Dignity may be defined as possessing an inherent value and worth.^2^ It is individual and situational,^5–7^ and is promoted or violated in situations where culture and environment are crucial.^8–10^ Experiencing dignity is fundamental for all people.^11,12^ It contributes to increased health and quality of life,^13,14^ is essential for satisfaction and well-being, enables good relationships and creates conditions that provide growth and development.^7,11,15,16^

The literature distinguishes between having dignity and being given or treated with dignity.^6^ Gallagher^17^ describes this as a self-regarding and other-regarding value. While other-regarding value is about respecting the dignity of others, self-regarding value is about respecting one’s own. Respect for one’s own dignity depends on one’s own worth and value being recognized. This subjective dignity is a personal experience, which Haddock^6^ describes as the ability to feel important and valued in relation to others. This aspect has been given less attention and is the aspect this article will address.

In education, the experience of dignity is an under-communicated topic.^18,16^ Increased awareness of dignity in nursing education can improve the formation process and the professional development for students.^19^ Research shows that students' experience of dignity can contribute to increased learning, increase their sense of self-worth and capablility.^20^ Experiencing dignity also helps to recognize and promote the dignity of others.^21,17^ In general, the experience may promote security and control,^10^ give the feeling of being empowered and able to make decisions,^22^ and provide motivation and self-confidence.^3^ Conversely, lack of experience of dignity may contribute to insecurity, guilt and shame, frustration, stress and anxiety, be demoralizing and demotivating,^3,23^ and result in poorer self-esteem and self-respect.^2^

In general, the goal of education is to promote student dignity.^24^ Nevertheless, perceived dignity is relative and shaped by relationships.^8^ The relationship with the supervisors will be crucial for the students, both in the educational institutions and in the clinical field. Gillespie^25^ calls the student–supervisor relationship the “arena of opportunity,” to emphasize the potential that lies in the relationship. In this article, the student–supervisor relationship will include both the students’ relationship to academic supervisors and clinical supervisors.

Research on nursing students shows that dignity is at stake. In summary, the research field provides examples of students experiencing disrespectful treatment,^26–28^ or not feeling recognized and prioritized.^27,29,30^ These experiences are evident both in educational institutions^26^ and in the clinical field.^27^

In addition to research on aspects of the student–supervisor relationship,^31^ research on students' experiences of their own dignity is in demand.^3^ Based on a lack of research and our curiosity about how nursing students experience their education, this study aims to explore how nursing students experience their own dignity during education. The study’s research questions are:

How do nursing students experience their own dignity in relation to supervisors, and what significance do these experiences have in their education?

## Theoretical framework

The study’s findings will be discussed in the light of Eriksson,^32,33^ Edlunds^34^ and Edlund et al.’s^8^ understanding of dignity, as well as Løgstrup’s^35–39^ and Martinsens^40–42^ reasoning about human relations. They all understand life as created and given, something people receive, at the same time as people relate to each other and create each other’s existence through irrevocable and irreversible actions. Such situations are described as a tilting position. The condition, and consequently the outcome, may tilt one way or the other. Humans are created relational, and according to Løgstrup being given sovereign and spontaneous expressions of life, such as trust, openness of speech, mercy, compassion, sincerity and hope, which are fulfilled in a situation.^35,37,39^ In human encounters, these expressions of life are put at stake. Either man may be open to them and fulfill them, or fail them. If they fail, human relationships are destroyed.^40,41^

Like the sovereign and spontaneous expressions of life, the absolute dignity is given to all human beings. It is constant and inviolable.^8,32–34^ At the same time, dignity also has a relative side. The human experience is shaped in encounters with others. The dignity a person experiences is influenced by how much space the sovereign and spontaneous expressions of life is given in these encounters. The relative dignity, which is changeable, can be violated and rebuilt. It is experienced in relationships and only becomes valid when people encounter people who convey it.^8,32–34^ Humans are each other’s worlds and destinies. They hold each other’s lives in their hands and are thus at the mercy of each other. They surrender themselves to each other, in the confidence of being received.^37^ Sensing and open, they receive each other, becomes affected and their minds and interactions becomes attuned. The attunement will be the undertone of everything people are and do.^40–42^ The sensing attunement gives spiritual nourishment and courage of life. It gives humans the courage to venture forward to live and receive.^40^ Human encounters can set an attunement that evokes life, or causes a person to wither, ease a sadness, or amplify it. It may also be about a frightening amount of issues, such as being responsible for whether one’s neighbor succeeds or not.^37^

The relative dignity contains an inner ethical form, which includes the morality and the norms that humans have made their own. It is expressed, among other things, as pride and independence. The external esthetic form of relative dignity serves as an arena where dignity is expressed through actions and attributes, for example by showing restraint and decency. Dignity is experienced when humans experience harmony between their own abilities, knowledge and the demands they have on themselves, or others have on them.^8,34^ To experience dignity, a person needs human encounters where dignity can be expressed and affirmed. Man understands himself from his surroundings and lives off the possibilities of identification that the surroundings offer. Without these, man cannot become himself.^36^

To not be accepted is to deprive man of the courage to live. This may encompass being met with indifference, reservation and rejection. Man makes himself vulnerable by exposing himself and reacts strongly when trust is abused. The consequences may be that man never again ventures forward, or criticizes and blames himself.^35,37^ When the sovereign and spontaneous expressions of life do not have room to be fulfilled, an ethical demand arises. It demands that we take care of our neighbor. This ethical demand wants to be superfluous. That can only happen if the sovereign and spontaneous expressions of life are fulfilled and thus make it superfluous. The ethical demand is formulated as the golden rule. Do unto others as you would have them do unto you. The ethical demand is silent about what this means in encounters with others. To gain more insight on this one must use one’s insight, imagination, judgment, and understanding.^35,37^

## Methodology

### Design

The study seeks to increase the understanding of nursing students' experiences of their own dignity in education, as expressed in narratives and transcribed interviews. Gadamer’s philosophical hermeneutics was chosen as the approach. According to Gadamer,^43^ understanding comes through interpretation, and the process of interpretation is dialectical. When something unforeseen is discovered in a text, the art is to be open to the text’s uniqueness, what it really means, and ask about the underlying factors. Through questions, phenomena become visible, but the questions are limited by the horizon one sees from. What becomes visible from this field of vision is determined by the situation, historical reality and associated tradition. This constitutes the individual’s pre-understanding, which is also the condition for understanding. With the help of pre-understanding, the horizon of understanding can be expanded. This happens through the hermeneutic circle. An interpretive movement between whole and part, between what is examined and the context in which the interpretation takes place, and between human pre-understanding and what is examined. The whole is understood on the basis of the parts and the parts on the basis of the whole. If a person encounters something unexpected or something that breaks with their own pre-understanding, the pre-understanding is put at stake and corrected. Meaning springs out through merging of horizons, where expansion of horizon takes place.^43^

### Participants and research context

The study has a strategic accessibility sample, which means that all students who met the inclusion criteria and who made themselves available were included. The sample consists of 19 nursing students, 15 women and four men, in their third and final year of nursing education, and they are aged 21–37 years. They represent six different education campuses in Norway, where both universities and university colleges are represented. The educational institutions were of different sizes and followed different curricula and syllabi. The students received written and oral information about the study after access to the students was given by the educational institutions. All interested students signed a declaration of consent and were included in the study.

### Data collection

Nineteen individual interviews were conducted. The interviews were conducted in suitable rooms at the students’ place of study. Prior to the interviews, all students presented either a written or oral narrative, about a time they experienced, possibly not experienced, a sense of dignity during the nursing education. The narratives were used as a supplement to the interviews to generate richer and more detailed descriptions. Narrative can help to bring out the complexity of experiences.^44^ Through stories, dignity can become clearer. It can be evident through examples of what dignity can be in the current situation.^45^ Through the students' stories, we gained insight into how they understood dignity and what was important to them. Together with the narratives, which served as a starting point for the interviews, the researcher used a conversation guide. Examples of topics in the conversation guide were dignity as a phenomenon, how dignity is maintained and violated, and what significance experienced dignity may have. The researcher tried to create a safe atmosphere where the students felt heard and confirmed. On average, the interviews lasted 76 min. Nonverbal observations were noted, and all interviews were recorded on audiotape and transcribed verbatim.

### Data analysis and interpretation

In addition to Gadamer’s^43^ statements on how understanding is achieved, the study’s interpretive process is inspired by Flemming, Gaidys, and Robbs’^46^ interpretive steps. The narratives were included in the transcribed interview text and interpreted in the same way as the rest of the data material.

In order to raise awareness of one’s own pre-understanding, both before and during the interpretation process, one’s own pre-understanding was written down and in thereby made available for reflection and discussion.

The audio files were listened to, and the transcripts read, several times. In this way, an overall impression emerged which was written down and which was the starting point for how the parts of the data material were understood further.

Through a dialectical process with the text and the context of the interviews, meaning units were identified and themed. All meaning units with related topics, from all the interviews, were read together. In this reading, different nuances of the themes were identified as sub-themes. Identification of topics and sub-topics was made on the basis of discussions in the research team. The theme and sub-themes were abstracted by asking what these were most deeply about, and constantly mirrored against one`s own pre-understanding and overall impression. This resulted in two main themes, where one main theme corresponds to how the students experienced their own dignity in relation to supervisors, and one that sheds light on the significance of these experiences in the education. (Table 1)

**Table 1.** Example of the interpretation process.

Overall impression	Meaning units	Sub-themes	Main themes
The experience of dignity is fragile	«I really felt seen, not only as a student, but also as a human being. I had some personal problems at that time, and the teacher took time with me, listened and gave advice. It meant a lot to me, because it helped that I did not feel completely lost” (5)	To be seen	Dignity at stake—The crucial importance of confirmation
	«In the morning when I came in, she did not say hello to me. (…) She went straight into the meeting room, where we distributed work tasks (…), and then she left the room without me. It could take a quarter of an hour and then she could come back and just say; oh-yes you are here today. Are you with me today?” (6)	To be ignored	
The importance of being confirmed or not confirmed	«The experience of dignity made me feel that; ok maybe I can be a nurse. Because I have in a way had my uncertain times where I have somehow thought that this seems too tough, too difficult, too hectic. But there I got a feeling that ok I can master this and with a little more practice and time I can manage this on my own as well» (5)	To be encouraged	Reactions to perceived confirmation or lack thereof
	«I kind of started thinking that maybe I’m not fit, maybe I’m not good enough. I have gone all the time and felt far too confident that I am capable and that I am good enough, but suddenly I get feedback that I may not be. Then you start to reconsider that you may have made the wrong choice. Maybe I should not work with people at all. I just had to work very hard with myself afterwards. I had to work with the psyche and I had to work with my attitude» (8)	To be discouraged	

### Ethical considerations

The Declaration of Helsinki^47^ was indicative throughout the research process. The students signed an informed consent form, and voluntary and continuous consent was emphasized. They were informed that participation was voluntary and that participation would have no bearing on their further studies. It was emphasized that the researcher has a duty of confidentiality and was not affiliated with any of the educational institutions. In some interviews, the students shared stories about painful and degrading situations. The researcher reflected on one’s own research role in encounters with the students and discussions were made in the research team on how the researcher should relate to this. The researcher sought to meet students with empathy and understanding. It was ensured that the students had someone to talk to, and the researcher also contacted them afterward, all in accordance with the students. Possible strains were assessed against what it gave the students to participate. The audio tapes were deidentified by transcription. Student names were changed to the numbering 1–19, and consent forms, audio files and connection keys were stored in accordance with regulations. The study was approved by The Norwegian Center of Reporting Data (NSD), and current guidelines were followed.

## Findings

The interpretation of the empirics resulted in the following two main themes 1) Dignity at stake—The crucial importance of confirmation, and 2) Reactions to perceived confirmation or lack thereof. How the main findings answer the study’s research questions about how nursing students experience and respond to dignity are presented below. The main findings are elaborated through dichotomies, depending on whether the dignity experience was present or not.

### Dignity at stake—The crucial importance of confirmation

The students experienced that dignity was at stake during their education. The experience of dignity was fragile, and they felt defenseless and at the mercy of their supervisors. The experience tipped between dignity and offense. How the students were confirmed in relation to academic and clinical supervisors was decisive. Below, the forms of confirmation that were most important for the students' experience of dignity will be presented.

#### To be seen as an individual or an object

In the students’ opinions, dignity was about being seen and understood as the person they were, with their history, their strengths and weaknesses, and being unconditionally accepted. It was important that the supervisors took the time to look behind the facade to get to know them.

“I felt seen. So when I talked about things, the clinical supervisor asked very open questions to find out more, because it seemed like she was genuinely curious about how I was doing. She made me feel visible and that was good.” (5).

The students experienced being seen when they were treated individually in different ways. They experienced that their uniqueness was taken seriously and that they were given space to be themselves. It was especially important to be met on individual needs in the learning process. Although the education as a system was perceived as rigid, in terms of individual adaptations, the students experienced that the academic supervisors in particular, used discretion to challenge the system and find ways around, if necessary.

The contrast to being seen as an individual with individual needs was to be seen as one in the crowd and met with little individuality. Several students missed the experience of being seen as they were, especially in relation to clinical supervisors.

“It was very odd to walk around and be called either you or the student all the time. I’m sure the clinical supervisor did not even know my name. I did not feel very welcome and seen. (…). The name is me, it’s my identity. I’m Natalie, not the student. The student becomes so foreign. Everyone can be a student, but only I am Natalie.” (6).

Other students had experienced being categorized and stigmatized as a group. For example, the useless, lazy or incompetent students.

#### To be acknowledged or rejected

Many experienced to be acknowledged when they were respected as human beings, included, taken seriously and listened to. They had to experience in words and deeds to be given value.

“For me, dignity means feeling respected and heard. For example, if we are sitting around a table; okey, now it’s your turn to talk. That everyone is included and heard. It’s very important to me.” (19).

The students also experienced acknowledgment when someone spent time with them and made an effort. This was interpreted as a sign of them being worth investing in and deserving the best. They had experienced academic or clinical supervisors who showed a great deal of commitment, presence and who followed them closely. This was especially important when the students had challenges and needed care, support or extra facilitation.

The opposite was to be rejected and met with indifference, something several had also experienced. They talked about the feeling of being treated like air. This was especially true in encounters with clinical supervisors.

“When we students came to the ward on the first day of practice, we had to try to find someone who told us who the clinical supervisors were. When I and another student entered the room, three nurses were sitting with their backs towards us. Nobody turns around and says hello, but I say: Hi, I think you are my clinical supervisor? She who was supposed to be my clinical supervisor turns and looks at me, she then turns back to the PC while she says; I’ll talk to you afterwards. When the other student standing there asks if her clinical supervisor is there, the other nurse breaks out; students this year too, I actually do not bother, why do I or our group always have to have those students.” (1).

Others had experienced clinical supervisors who left them behind and did not inform them of where they went, and/or changed their shifts to avoid supervising.

Lack of acknowledgment that the students' knowledge was valuable to the workplace was also prominent. Several students had experienced being neglected, not taken seriously, being ridiculed or called stupid, when they tried to contribute with their knowledge.

#### To be reassured or made insecure

The students were vulnerable during education. Constantly meeting new challenges was demanding, and the need for security, predictability and control was prominent. Dignity was linked to self-confidence and one’s own abilities. The security was built up through repeated experiences of being given responsibility and experiencing control and mastery. In particular, it was important to have the time, acceptance and space to present oneself as inexperienced, fallible, and honest. Clear requirements and expectations, and thorough and understandable feedback also promoted security and control. To have both academic and clinical supervisors who supported them when they felt insecure, cheered them on, and showed them confidence, was absolutely essential.

“When I enter a new arena, I have not felt safe, but I have felt confident that this is going well because the clinical supervisor is involved and she believes that I can do it and help me if I cannot do it (…). If she has confidence that I can make it happen, then the confidence is not unfounded (…). Then she sees something I do not see, and then I feel more secure and comfortable and dare to prove myself.” (5).

Losing confidence and self-esteem was perceived as losing ground. Many rarely went into situations they were unsure of. Losing face was risky. Several students experienced the clinical supervisors' daily form and mood swings unpredictable. Some were met with a “failed” clinical study, without any kind of warning.

“I felt insecure when I experienced this coming like lightning from clear skies. I felt secure, but maybe I did not have any reason for feeling secure. After this, I have been very skeptical at every clinical practice, and it has only escalated in all the other practices where I have been really insecure. You are afraid that the same thing will happen again, that you really have no reason to feel secure.” (8).

The experience of unpredictability, insecurity and incapacitation could also have its background in degrading situations. Some had been blamed in the presence of patients, relatives and other healthcare professionals, had been scolded, forced to make procedures wrong and left to fend for themselves in situations they were unable to cope with. Some had also experienced behavior that was perceived as sexual harassment.

### Reactions to perceived confirmation or lack thereof

Experiences with dignity and violations of dignity left their marks in the students and gave different and contrasting reactions. The reactions highlighted the importance of perceived dignity and will be presented below.

#### Increased life courage or discouragement

First and foremost, the students said that perceived dignity gave them courage. They gained courage in the form of increased energy, optimism, motivation, positivity, well-being and quality of life. The students experienced themselves as safer professionals and practitioners, with increased confidence and better self-esteem. They became more resilient and described the experience as being able to carry themselves better in relation to others:

“You get a boost (…) Dare to go in, dare to be. To stand there, true. And be there.” (7).

If others recognized their value, it was also easier to have hope and faith in the future. The experience of dignity gave pride. The dignity experience gave pride in mastering the education and becoming part of the nursing profession. It contributed to the development of identity as nurses.

Lack of experience of dignity gave rise to feelings of shame, depression, insecurity, anger, frustration, confusion, and shock. They felt worthless, weak, stupid, small and useless. Lost was the self-esteem and belief in coping with the profession they were trained for. Lack of confirmations was experienced as a life crisis, and they lost hope and motivation. They described events as “the worst weeks of life” and as a feeling that “the whole universe was falling apart”. On some occasions, the experiences were compared with previous postpartum depression and depression after losing their parents. The experiences could also have physical effects:

“I left work an hour and a half earlier because I was so ill. I was nauseous, my stomach hurt a lot, and then I started crying. I felt I had nothing to do there. So I lay down in bed at home and cried because it was so uncomfortable.” (4).

The students described fatigue, tiredness and exhaustion. The experiences were bodily and lasted a long time. Two years after the negative events, some were surprised that they were still crying over this, or still feeling exhausted.

Instead of being proud of the nursing profession, several students became disillusioned. They lost respect for the profession, the education and the supervisors, whom they were supposed to look up to. They stopped believing in them and what they conveyed. The experience of powerlessness meant that they did not report the injustice they experienced. Capitulation, defenselessness and voicelessness were prominent.

#### Increased presence or absence

The experiences also led to the students either becoming more present in mind or absent. With the experience of dignity, the students dared to be present in relationships and situations. Presence in mind made it easier to be yourself, be in the now, be active, participate, focus on learning, and perform better. It also gave motivation to get involved, to complete the study and to dare to show confidence.

“It’s really just that I become more confident and comfortable and dare to show what I know.” (5).

Increased presence provided opportunities for learning and development. The students became more aware of themselves and it became clear to them who they wanted to be. Several also described that the ability to be present in patient situations became easier.

Lack of affirmations and violated dignity made the students withdraw both physically and mentally, and they wanted to be invisible. They “shut down” for learning and withdrew into themselves.

“When I was in clinical practice, I just disconnected completely. (…) That was what I had to do, because I was completely destroyed. I was in a bubble just for myself. I did not contribute much. Felt I was not thinking much about what I was doing, just went on the autopilot.” (4).

The result was that these students received feedback that they were apathetic, unresponsive, restrained, quiet, or took too little initiative, which made the situation even more challenging. The withdrawal was also expressed in a lack of commitment and participation in professional discussions. Some were absent from study groups, avoided clinical supervisors by changing shifts, asking to go with others or work alone. The withdrawal became especially visible when the students chose to stay at home from the educational institution or their clinical practice, because the conditions were too demanding. The ultimate withdrawal, however, was to end the education. This was something several had considered.

## Discussion

The main finding of the study is that the dignity of nursing students is at stake in encounters with supervisors in nursing education. Experiences with dignity and violation put the students' relative dignity in a tipping position. Factors surrounding the vulnerability, confirmation, and importance of dignity will be discussed here.

### The vulnerability of dignity

All human beings are given an absolute, constant, and inviolable dignity. Contextual circumstances can nevertheless overshadow absolute dignity, making it difficult to discover its existence.^34^ What remains is the relative dignity. The relative, changeable dignity is socially rooted, and the environment can both promote and violate it.^8^ The students had experienced this. It was experienced as a game of chance whether the surroundings took care of them or not. The students perceived their dignity as threatened, fragile and unprotected. The experiences varied from fantastic to completely destructive, and the dignity came in a tipping position. According to Løgstrup,^35^ every human encounter is at a tilting position. Either the sovereign and spontaneous expressions of life such as trust, openness of speech, mercy, compassion, sincerity and hope, are made room for in the relationships, or they are not. The students experienced dignity when the sovereign and spontaneous expressions of life were given space in the relationship with the supervisors. When the expressions of life did not take place, the students experienced mistrust, closedness, ruthlessness, insensitivity, dishonesty, and hopelessness. This marked their experience of dignity.

Constantly being measured and assessed made them feel vulnerable and the experience of dignity was threatened. The students had to experience harmony between their own abilities, knowledge and the demands they had on themselves, or that the supervisors had on them, in order to experience dignity. They experienced an inner ethical dignity if they managed to live up to the standard that was applicable in the culture and the context that the educational institutions and the field of practice represented. The students had to show that they had the necessary qualities to fit in. This gave the experience of pride and independence, which was essential for the experience of dignity. Through the external esthetic dignity, it became essential to have room to express symbols of one’s own worth, so that the academic and clinical supervisors could recognize and confirm these. This could be, for example, to perform actions that expressed restraint or orderliness in different situations. But this made the students vulnerable. They had experienced shame more than pride, dependence more than independence, shown insecurity more than restraint, and felt more useless than valuable. In these situations, they experienced their own dignity violated. This became especially apparent when they were below average in the learning process, were at risk of failing their clinical study, or when others called them incompetent, useless, lazy, and stupid. They needed the supervisors to receive them, identify and confirm the person they wanted to be, and the actions they took to substantiate this.

### The confirmation of dignity

Humans are involved in relationships with each other and affects and tune each other in these encounters.^40–42^ The students were at the mercy of their supervisors, and were affected and tuned by how the supervisors met them. The attunement told the students something about their value and influenced their experience of dignity. It set a tone and mood that could be healing or detrimental to health. By mirroring themselves in their supervisors, the students either experienced having their dignity confirmed or violated. Løgstrup^37^ describes this dependence on each other as carrying some of the other’s life in their hands.

In the students' approach to the supervisors, a tone was struck and in the tone was a silent request to be accepted. The claim was accepted if the students were confirmed. Humans need someone who conveys dignity to them. Edlund^34^ calls it a public approval of dignity, which means that it is perceived as valid. The confirmation is about having their worth confirmed. This is of particular importance in vulnerable and degrading situations,^34^ as the students had experienced several times. The most significant confirmations were to be seen as individual, recognized, respected, taken seriously, listened to, invested in and reassured. This is also supported by clinical research in the field.^10,22,^ Furthermore, Parandeh et al.^3^ found that respect for students’ individuality, knowledge, rights, choices, development and learning abilities was important.

It was crucial for the experience of dignity that the students were seen and accepted. That their name was remembered and that the uniqueness of them was accepted and acknowledged. It was also important to be seen as a resource and assigned value. Through the sense of being someone and feeling significant, dignity becomes noticeable.^34^ This experience did not occur when the students were objectified as one in the crowd, seen as a burden and as an insignificant one that could easily be replaced. Eriksson^48^ points out that not being accepted and acknowledged can cause suffering in humans. A person can then get a feeling of being non-existent to the other.

The students' everyday study consisted of several degrading situations, lack of control and unpredictability. Being met with trust and security therefore became especially important. According to Von Post,^49^ dignity can be evoked through touch, speech, embrace and glance. How the individual supervisor, for example, spoke to, or referred to the students, could confirm a security in the students that gave a foothold in an otherwise insecure learning situation. The relationship created between student and supervisor is of great importance, both for students' educational experiences, learning,^50^ potential achievement^25^ and development of nursing identity.^31^ The aim of the supervision is to support the students’ learning process towards becoming caring nurses. This starts with the students themselves feeling cared for through confirmation.^51^

### The importance of dignity

Supervisors have the students' courage and zest for life in their hands. The experience of dignity is vital and the basis of everything else. It is about succeeding or failing as a student. The students described that they gained courage when their dignity was confirmed. Life courage gave hope, joy, energy, motivation, quality of life, pride, and security, both personally and professionally. According to Løgstrup, the tone that is set in a relationship can give spiritual nourishment and life courage, give people the courage to venture forward to live and receive.^40^ This is also shown in the findings, in that perceived dignity gave increased presence. To be present had a bearing on the learning situation, on the learning as a whole, on the relationship with patients, and on whether they wanted to complete the study or not. The students dared to a greater extent to be guided, moved and developed.

The tone of the relationship can bring life and ease sadness, but it can also make people wither, intensify the sadness and make the other fail.^37^ Violated dignity led to hopelessness and withdrawal. Not only did they lose faith in themselves and their lives as nurses, but also in the nursing profession. They were often ashamed and wanted to hide. In their retreat, they were not able to show who they were and what abilities they had or lacked. The opportunity to reach their potential was weakened and they were not given the opportunity to express their own dignity.

The students' experiences of being met with indifference and rejection meant that the students did not dare to come forward again. The exposure became too demanding. Reactions to the violations varied. How people experience a breach of trust has to do with the human will and personality.^37^ Edlund^34^ points out that only the individual themselves knows what is offensive or not. This depends on what these individuals build their dignity on.

It is the supervisor who is responsible for creating a relationship, where students avoid being ashamed and who facilitates development and learning.^52,53^ Human encounters are irreparable and irreversible.^37^ This emphasizes that relational competence must be prioritized and improved in education.^25^ Research shows an increasing problem with incivility in nursing education.^54–56^ Incivility is described as an attack on human dignity, as destructive to human self-esteem and experience of self-worth, and as something that has negative consequences for human mental and physical well-being.^54^ Furthermore, it hinders professional development.^57^

Promoting essential values in the nursing profession is the supervisor’s responsibility.^57^ Violation of other people’s dignity can occur both consciously and unconsciously.^34^ When encountering a student, it can be difficult to know how to promote his dignity. How this can be done, is not obvious. It depends on the situation. A good rule of thumb, however, is to meet the students as you yourself would like to be met in a similar situation, as the golden rule dictates.^37^ Living by the golden rule is perhaps the most effective approach in confirming another person’s dignity.^32^ This requires a willingness to use insight, imagination, understanding and judgment in encounters with the students. In this way, good conditions for confirming students’ dignity are created.

## Strengths and limitations

The study provided no guidelines for how dignity should be understood. Through narratives, the students described their understanding of the phenomenon. The authors tried to be conscious of our pre-understanding so that it did not subconsciously affect our interpretations. The pre-understanding was put into play in the face of empiricism. Increased insight into the concrete meaning of dignity and nuanced expressions provided expanded understanding.

The students represented different educational institutions with their respective learning environments and curricula. This may have influenced the students’ experiences. It is also conceivable that the students who chose to participate in the study did so because they either had very positive or negative experiences with dignity in the education.

## Conclusion and implication for practice

The study reveals that students’ dignity is at stake in encounters with supervisors during education. Decisive for the experience of dignity was how the students experienced that the supervisors in different ways confirmed them. Perceived dignity gave them the life courage and increased their ability to be present, which they needed as students and future nurses.

It is essential that the academic and clinical supervisors have time and frameworks that create a basis for following up the students professionally and personally, so that the students can feel valued. It will also be important that the educational institutions and the clinical field work together to develop reflected educational and learning cultures that emphasize that students should experience the supervisor’s commitment, care and appreciation. The students will then be enabled to pass this on to patients and relatives, and in turn to new nursing students whom they themselves supervise.

All human beings are created equal in dignity. However, the dignity becomes relative in human relationships. In all human relationship’s dignity becomes vulnerable as it can either be confirmed or degraded. As individuals, we must take responsibility for making the other’s world as spacious as possible, and make room for values that symbolize the worth of “being” rather than “doing.” We might say that if a human`s absolute dignity is understood and respected, the relative dignity also has better opportunities to be protected. If the sovereign and spontaneous expressions of life are given space among us, we also give room for confirmation of the dignity of individuals.
